# Supplementary material for: 2-Imidazoline Nitroxide Derivatives of Cymantrene
Source: Molecules. 2022 Nov 3;27(21):7545. doi: 10.3390/molecules27217545 (PMC9659262; doi:10.3390/molecules27217545)
Supplement: Supplementary file 1 [file molecules-27-07545-s001.zip › molecules-1990285-supplementary.pdf]

SUPPLEMENTARY MATERIALS

2-Imidazoline nitroxide derivatives of cymantrene

Kseniya Maryunina [1,*], Gleb Letyagin [1,2], Galina Romanenko [1], Artem Bogomyakov [1,2,3], Vitaly Morozov [1], Sergey Tumanov [1,2], Sergey Veber [1,2], Matvey Fedin [1,2], Evgeniya Saverina [3], Mikhail Syroeshkin [3], Mikhail Egorov [3] and Victor Ovcharenko [1,3,*]

[1] International Tomography Center SB RAS, Institutskaya Str. 3a, 630090 Novosibirsk, Russia

[2] Novosibirsk State University, Pirogova Str. 1, 630090 Novosibirsk, Russia

[3] N. D. Zelinsky Institute of Organic Chemistry RAS, 119991, Leninsky Prospect, 47, Moscow, Russia

*Correspondence: mks@tomo.nsc.ru (K.M.); Victor.Ovcharenko@tomo.nsc.ru (V.O.)

Table S1. Crystal data and experimental details

Compound	**NNMn**	**INMn**
FW	359.24	343.24
T, K	296	240
Space group, *Z*	$P2_1/n$, 8	$P2_1/n$, 8
a,<br>*b*,<br>*c*, Å	16.7512(10)<br>10.1789(5)<br>20.7153(11)	16.5699(7)<br>9.9203(5)<br>20.5093(9)
α,<br>β,<br>γ, °	90<br>111.278(4)<br>90	90<br>109.432(2)<br>90
V, Å^3	3291.4(3)	3179.3(3)
D_c, g cm^{-3}	1.450	1.434
θ_{max}, deg.	28.299	67.986
I_{hkl} (meas/uniq)<br>R_{int}	28169 / 8170<br>0.1200	33501 / 5724<br>0.0482
$I_{hkl}(I>2\sigma_I)$ / *N*s	2607 / 439	5032 / 498
GooF	0.807	1.043
R_1 / wR_2 ($I>2\sigma_I$)	0.0472 / 0.1018	0.0366 / 0.0979
CCDC	2182686	2182685

Table S2. Selected bond lengths (Å) and angles (°)

Compound		M–C_{CO}	M–C_{Cp} (M–centroid)	N–O	∠Cp–{O●-N-C=N(→O)}	C_{Cp}–C_{NN}
NNMn	**A**	1.749(7), 1.783(6), 1.789(7)	2.133(6)–2.155(5) (1.776)	1.276(5), 1.271(5)	5.3	1.437(6)
	**B**	1.767(7), 1.786(6), 1.787(6)	2.130(6)–2.149(4) (1.771)	1.272(4), 1.273(4)	19.8	1.435(6)
NNRe-III[1]	**IIIA**	1.869(11), 1.921(12), 1.911(11)	2.290(8)–2.311(8) (1.958)	1.282(9), 1.281(8)	4.6	1.483(11)
	**IIIB**	1.870(12), 1.923(11), 1.920(10)	2.289(8)–2.303(8) (1.953)	1.277(8), 1.285(8)	30.3	1.450(11)
INMn	**A**	1.789(3), 1.798(3), 1.803(3)	2.118(3)–2.148(3) (1.763)	1.240(5) 82%	6.0	1.462(4)
	**B**	1.792(3), 1.796(3), 1.807(3)	2.130(2)–2.145(2) (1.766)	1.265(3)	13.8	1.457(3)
INRe[1]		1.893(8), 1.903(6), 1.912(7)	2.294(5)–2.315(6) (1.958)	1.205(6)	7.4	1.452(7)

Table S3. Selected intermolecular distances (Å) and angles (°); the distances that can give the most significant contribution to the formation of magnetic exchange interactions channels are highlighted **in bold**.

Compound		$O_{CO}…O_{NO}$	$C_{Cp}...C_{Cp}$ centroids	∠Cp…Cp	$C_{Cp}…O_{NO}$	$O_{NO}…O_{NO}$	$N…O_{NO}$	N…N
NNMn	**A-A**	4.190(7) 4.196(6)	–	–	**3.24(1)**	–	–	–
	**A-B**	-	3.292(8)	20.0	3.623(7)	**4.125(5)**	**3.956(5)**<br>**4.007(5)**	**4.079(5)**
	**B-B**	4.14(1)	–	–	–	–	–	–
NNRe-III[1]	III**A**-III**A**	4.22(1) 4.06(1)	–	–	**3.20(3)**	–	–	–
	III**A**-III**B**	–	3.24(1)	20.4	3.60(1)	**4.07(1)**	**3.93(1)**<br>**3.94(1)**	**4.04(1)**
	III**B**-III**B**	4.14(1)	–	–	–	–	–	–
INMn	**A-A**	4.459(4)	–	–	**3.304(5)**	–	–	–
	**A-B**	–	3.323(3)	23.1	3.441(4)	–	–	**4.301(3)**
	**B-B**	–	–	–	–	–	–	–
INRe[1]		3.95(1)	3.33(2)	0.0	3.12(1) 3.17(1)	**3.69(1)**	**4.55(1)**	5.539(6)

Table S4. The intermolecular magnetic exchange-coupling parameters (cm^{-1}) according to results of analysis and fitting of magnetochemistry data (fitted) and periodical quantum-chemical calculations (calculated; Quantum Espresso 6.2 package,[2] PBE+U).

	fitted				calculated		
	J	***g***	**monomer impurity**	spin Hamiltonian[3]	***J***	contact type	spin Hamiltonian[3]
NNMn	-12.4	2.01	0.01	$H = -2JS_{R1}S_{R2}$ $S_{R1} = S_{R2} = ½$	-3.0 (-3.15)*	NO…ON	$H = -2JS_{R1}S_{R2}$ ($S_{R1} = S_{R2} = ½$)
					~0 (-0.01)*	Cp…ON	$H = -2J_{\text{chain}}\sum_{i=1}^{n-1} S_{R_i}S_{R_{i+1}}$ ($S_{Ri} = ½$)
NNRe-III[1]	-12.1	2.02	0.02	$H = -2JS_{R1}S_{R2}$ $S_{R1} = S_{R2} = ½$	-5.3	NO…ON	$H = -2JS_{R1}S_{R2}$ ($S_{R1} = S_{R2} = ½$)
					-2.0	Cp…ON	$H = -2J_{\text{chain}}\sum_{i=1}^{n-1} S_{R_i}S_{R_{i+1}}$ ($S_{Ri} = ½$)
INMn	-0.8	2.00	–	$H = -2JS_{R1}S_{R2}$ $S_{R1} = S_{R2} = ½$	–		

*These values were obtained by molecular DFT calculation[4] on the level TPSSh/ma-def2-TZVP.

Table S5. The calculated[4] Mulliken spin density values localized on atoms of {O←N=C–N–•O} or {N=C–N–•O} fragments and metal centers, g_{iso} and A_{iso}^{Ni} (mT) from modeling of EPR data, calculated[4] α-SOMO and β-LUMO orbitals energy values (eV), oxidation E_{ox}^{o} / $E_{ox}^{o}{}'$ and reduction potentials E_{red}^{o} (mV) from CV experiments for nitroxide-substituted cymantrenes and cyrhetrenes.

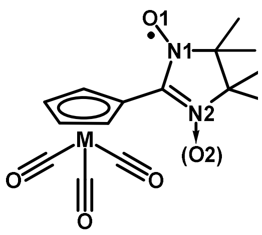

M = Mn, Re

Compound	Mulliken spin density M^{+}	Mulliken spin density O1 / N1 / C / N2 / O2	g_{iso}	A_{iso}^{N1} / A_{iso}^{N2}	α-SOMO	E_{ox}^{o} / $E_{ox}^{o}{}'$	β-LUMO	E_{red}^{o}
NNMn	-0.029	0.356 / 0.293 / -0.258 / 0.356 / 0.294	2.009	0.721 / 0.721	-8.068	824 / 793	-0.466	-1096
NNRe	-0.017	0.357 / 0.294 / -0.250 / 0.353 / 0.289	2.009[1]	0.726 / 0.726[1]	-8.103	713 / 681[1]	-0.505	-1041[1]
INMn	-0.012	0.492 / 0.336 / -0.114 / 0.284 / –	2.009	0.915 / 0.418	-8.560	1284 / –	-0.644	-982
INRe	-0.007	0.492 / 0.335 / -0.113 / 0.286 / –	2.009[1]	0.905 / 0.445[1]	-8.626	1270 / –[1]	-0.723	-952[1]

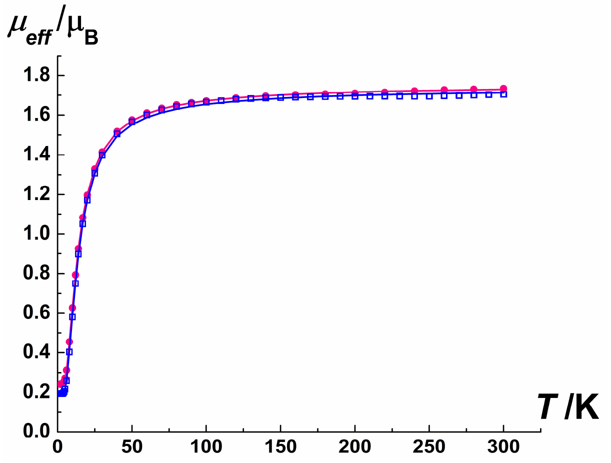

Figure S1. Experimental dependences μ_{eff}(T) for the nitronyl nitroxides NNMn (blue empty squares) and NNRe-III[1] (pink circles) and fitted curves (solid thick lines; Table S4).

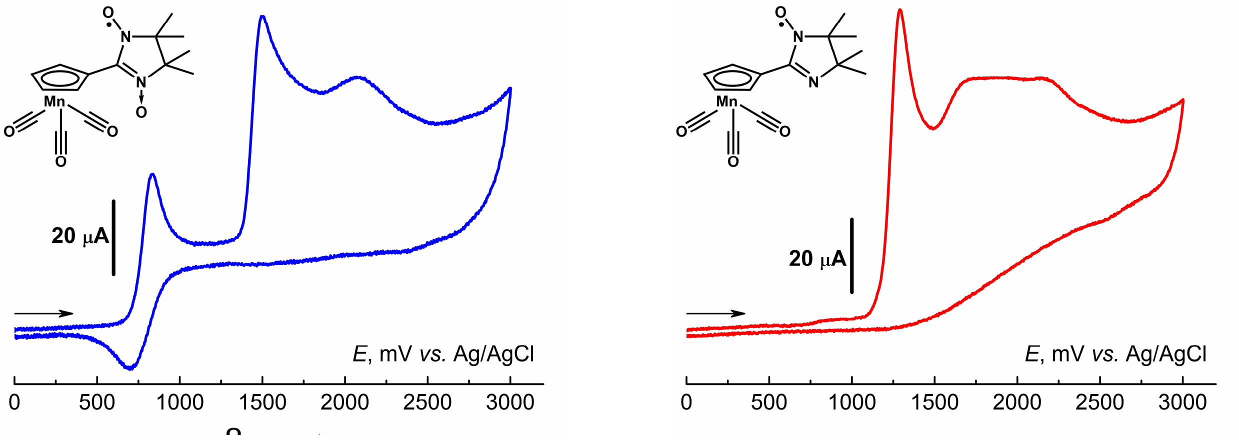

a ***b***

Figure S3. CV curves of oxidation of NNMn (a) and INMn (b); $5.0 \cdot 10^{-3}$M solutions in MeCN, a GC disk electrode *d* = 1.7 mm, supporting electrolyte 0.1 M Bu_4NBF_4/MeCN, the scan rate of application of potentials 100 mV·s^{-1}, *T* = 298 K.

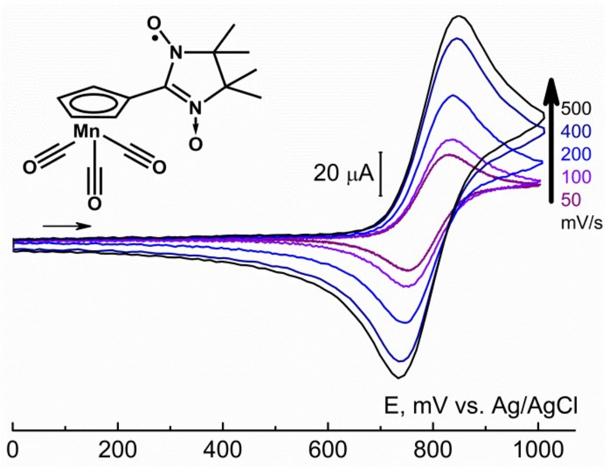

Figure S3. CV curves of oxidation of NNMn $5.0 \cdot 10^{-3}$M solution in MeCN at different scan rates of potential application (a GC disk electrode *d* = 1.7 mm, supporting electrolyte 0.1 M Bu_4NBF_4/MeCN, *T* = 298 K). The shape of the CV curves, the ratio of the currents of the anode and the cathode peaks and the apparent number of electrons (n_{app}~ 1) are independent on the 50-500 mV·s^{-1} scan rate of application of potentials.

REFERENCES

1. Maryunina, K.; Letyagin, G.; Bogomyakov, A.; Morozov, V.; Tumanov, S.; Veber, S.; Fedin, M.; Saverina, E.; Syroeshkin, M.; Egorov, M.; Romanenko, G.; Ovcharenko, V. Re(I)-nitroxide complexes. *RSC Adv.* **2021**, **11**, pp. 19902–19907, doi:10.1039/D1RA02159A.

2. Giannozzi, P.; Baroni, S.; Bonini, N.; Calandra, M.; Car, R.; Cavazzoni, C.; Ceresoli, D.; Chiarotti, G. L.; Cococcioni, M.; Dabo, I.; Dal Corso, A.; de Gironcoli, S.; Fabris, S.; Fratesi, G.; Gebauer, R.; Gerstmann, U.; Gougoussis, C.; Kokalj, A.; Lazzeri, M.; Martin-Samos, L.; Marzari, N.; Mauri, F.; Mazzarello, R.; Paolini, S.; Pasquarello, A.; Paulatto, L.; Sbraccia, C.; Scandolo, S.; Sclauzero, G.; Seitsonen, A. P.; Smogunov, A.; Umari, P.; Wentzcovitch, R. M. QUANTUM ESPRESSO: a modular and open-source software project for quantum simulations of materials. *J. Phys.: Condens. Matter*. **2009**, 21, pp. 395502, doi:395502 10.1088/0953-8984/21/39/395502.

3. Boča, R. *A Handbook of Magnetochemical Formulae*; Elsevier Inc., 2012, 1010 p.

4. Neese, F.; Wennmohs, F.; Becker, U.; Riplinger, C. The ORCA quantum chemistry program package. *J. Chem. Phys.*, **2020**, 152, pp. 224108, doi:10.1063/5.0004608.
